# Fluctuation-based imaging of nuclear Rac1 activation by protein oligomerisation

**DOI:** 10.1038/srep04219

**Published:** 2014-02-27

**Authors:** Elizabeth Hinde, Kyoko Yokomori, Katharina Gaus, Klaus M. Hahn, Enrico Gratton

**Affiliations:** 1 Laboratory for Fluorescence Dynamics, Department of Biomedical Engineering, University of California, Irvine, USA; 2 Department of Biological Chemistry, University of California, Irvine, USA; 3 Centre for Vascular Research and Australian Centre for NanoMedicine, University of New South Wales, Sydney, Australia; 4 Department of Pharmacology and Lineberger Cancer Center, University of North Carolina, Chapel Hill, NC, USA

## Abstract

Here we describe a fluctuation-based method to quantify how protein oligomerisation modulates signalling activity of a multifunctional protein. By recording fluorescence lifetime imaging microscopy (FLIM) data of a FRET biosensor in a format that enables concomitant phasor and cross Number and Brightness (cN&B) analysis, we measure the nuclear dynamics of a Rac1 FRET biosensor and assess how Rac1 homo-oligomers (N&B) regulate Rac1 activity (hetero-oligomerisation with the biosensor affinity reagent, PBD, by FLIM-FRET) or interaction with an unknown binding partner (cN&B). The high spatiotemporal resolution of this method allowed us to discover that upon DNA damage monomeric and active Rac1 in the nucleus is segregated from dimeric and inactive Rac1 in the cytoplasm. This reorganisation requires Rac1 GTPase activity and is associated with an importin-α2 redistribution. Only with this multiplexed approach can we assess the oligomeric state a molecular complex must form in order to regulate a complex signalling network.

Rac1 is a multi-functional protein best known for its roles in cytoskeletal organization and regulation of cell migration. Rac1 has long been identified as a key regulator in actin remodeling for cell ruffling, adherens junction formation and establishment of cell polarity^1^. More recently, Rac1 has been implicated in several nuclear functions, such as transcriptional activation^2^, cell cycle progression^3^, G2/M checkpoint activation^4^ and the DNA damage response^5^,^6^, yet the binding partners and mechanisms of Rac1 activity in the nucleus are largely unknown. By what means Rac1 achieves differential regulation of signaling in different cellular contexts is poorly understood. Increasing evidence suggests that subcellular location plays a major role in regulating the signaling output of promiscuous regulatory proteins such as Rac1^7^. Thus, to understand the multi-functional nature of Rac1 requires methodologies that can measure Rac1 activation and subsequent interaction with different candidate binding partners, preferably at different sub-cellular locations.

In live cells, detection of protein activity is most often accomplished by the use of FRET biosensors^8^,^9^,^10^,^11^. For example Kraynov *et al.* developed the biosensor Rac1 FLARE, a dual chain FRET biosensor which detects Rac1 activity based on the known interaction of active Rac1 (Rac1 bound to GTP) with a fragment of the effector p21-activated kinase 1 (PAK1)^12^. Protein activity has been shown in several aspects of cell biology to be regulated by protein oligomerisation. For example, Zhang *et al.* demonstrated that the activity of Rac1 is modulated by the reversible formation of monomer and homo-dimers in both the inactive (Rac1 bound to GDP) and active (GTP-bound) states and that Rac1-GTP dimers lead to self-stimulatory GTPase activity^13^. Thus having the means to assess the stoichiometry of the donor and acceptor of a FRET biosensor upon activation would be important to understanding the regulation of activity detected. Furthermore, given that Rac1 is a multi-functional protein, knowing Rac1 is active is not sufficient to understand what role Rac1 is playing in a given signalling pathway. We also need the capability to probe Rac1 interaction not only with the affinity reagent of the biosensor (e.g. the PAK fragment in the FLARE biosensor), but also its interaction with different unknown binding partners.

Here we establish a fluctuation-based approach to measure how protein oligomerisation modulates signalling protein activity through simultaneous use of the phasor approach to fluorescence lifetime imaging microscopy (FLIM) and cross Number and Brightness (cN&B) analysis. The phasor approach to FLIM is a fit-free method of analysis which can spatially map and quantify the degree of biosensor FRET in each pixel of an image or line scan as a function of time^14^,^15^,^16^. Number and Brightness (N&B) analysis measures the ratio between the variance and the average intensity to obtain the brightness of individual molecules and the stoichiometry of a molecular complex when extended to calculation of cross-variance^17^,^18^. By FLIM imaging of a FRET biosensor in a format that enables concomitant phasor and N&B analysis in each pixel of an image, we demonstrate that this method can determine the oligomeric state of the biosensor FRET donor, FRET acceptor and subsequent stoichiometry upon forming a complex, as well as verify the degree of signalling activity detected by FLIM-FRET or probe interaction with an entirely different binding partner through a cross variance analysis.

We use this multiplexed method to investigate the role of Rac1 activation in the DNA damage response pathway and how the oligomeric state of Rac1 affects the profile of this activation. This enables us to correlate Rac1 activity with Rac1 oligomerisation and thus assess if Rac1's oligomeric state affects signalling activity. We demonstrate the ability of cN&B to probe different binding partners - without designing a FRET biosensor for each interaction - to investigate the processes in which Rac1 is translocated between the nucleus and cytoplasm. Intriguingly we discovered from simultaneous phasor and Number and Brightness analysis of the Rac1 FLARE dual chain biosensor, a nucleus-wide activation of Rac1 upon induction of DNA damage that resulted in a redistribution of Rac1 between the nucleus and cytoplasm based on its oligomeric state. The nucleo-cytoplasmic shuttling of Rac1 during this redistribution is associated with importin-α: when Rac1 activation was inhibited upon induction of DNA damage, the nucleo-cytoplasmic transport of Rac1 and importin-α was deregulated during DNA repair. Thus this multiplexed approach to *in vivo* detection of protein activation is a powerful approach to reveal the oligomeric state a molecular complex must form in order to achieve correct regulation of a signalling pathway.

## Results and Discussion

### Concomitant fluorescent lifetime and cross number and brightness analysis of a dual chain FRET biosensor

**Figure 1:**
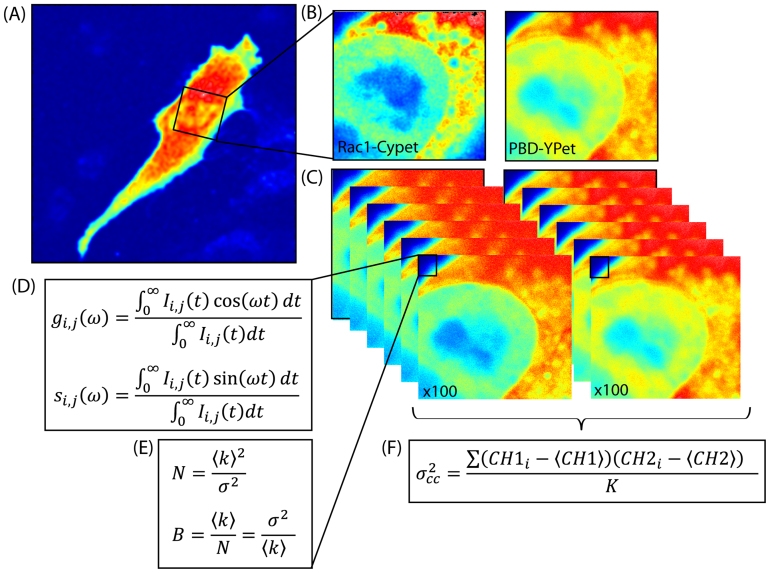
Biosensor FRET detection by concomitant phasor and cross number and brightness (NB) analysis. (A) Intensity image of a NIH3T3 nucleus transiently transfected with the Rac1 dual chain FLARE biosensor. (B) Intensity image of Rac1-CyPet (donor) and its binding partner PBD-YPet (acceptor) in a selected region within the NIH3T3 cell shown in (A). (C) Time series acquired of the same region as shown in B in the respective channels of Rac1-CyPet and PBD-YPet. (D) In each pixel of the frame acquisition we obtain a time series of the phasor coordinates (g_i,j_ and s_i,j_) with nanosecond resolution, which determines the pixel lifetime and reflects Rac1 activity. (E) In each pixel of the frame acquisition, we also obtain an intensity fluctuation with second resolution, which has a given average intensity (〈k〉) and variance (σ^2^) that describe the number (N) and brightness (B) of the molecules. (F) By calculating the cross variance in each pixel, we obtain the number of Rac1-CyPet molecules moving together with PBD-YPet.

To establish our multiplexed approach of analysis we measure simultaneously the fluorescence intensity and lifetime of the donor (Rac1-CyPet) and acceptor (PBD-YPet) of the Rac1 FLARE dual chain FRET biosensor transiently expressed in NIH3T3 cells (Fig. 1A–B). This was done with standard laser-scanning confocal microscopy using 2-photon excitation and time resolved detection in two separate channels (see Materials and Methods section). Using this set-up, we acquire a time series of frames in a region of interest of the cell (Fig. 1C) to obtain in each pixel of the selected frame a time series of the phasor coordinates (*g, s*) (Fig. 1D) which describe the pixel lifetime and an intensity fluctuation. Moment analysis of the acquired intensity fluctuations enables the number of molecules and their brightness to be extracted (Fig. 1E). Thus together the phasor and moment analysis enables us to compare the level of Rac1 activity detected by quenching of the donor lifetime (FRET) with the stoichiometry of the individual proteins and thus assess if oligomerisation of either chain affects Rac1 signalling activity. Critical to the success of this acquisition is the pixel dwell time. To obtain both lifetime and intensity fluctuation information we need to adopt a pixel dwell time that is long enough to integrate sufficient counts for lifetime determination but at the same time short enough to not average out the sample fluctuation. The interaction detected by FLIM-FRET can also be confirmed from a cross variance N&B analysis (Figure 1F), both of which provide pixel-resolution maps of the Rac1-PBD complex.

**Figure 2:**
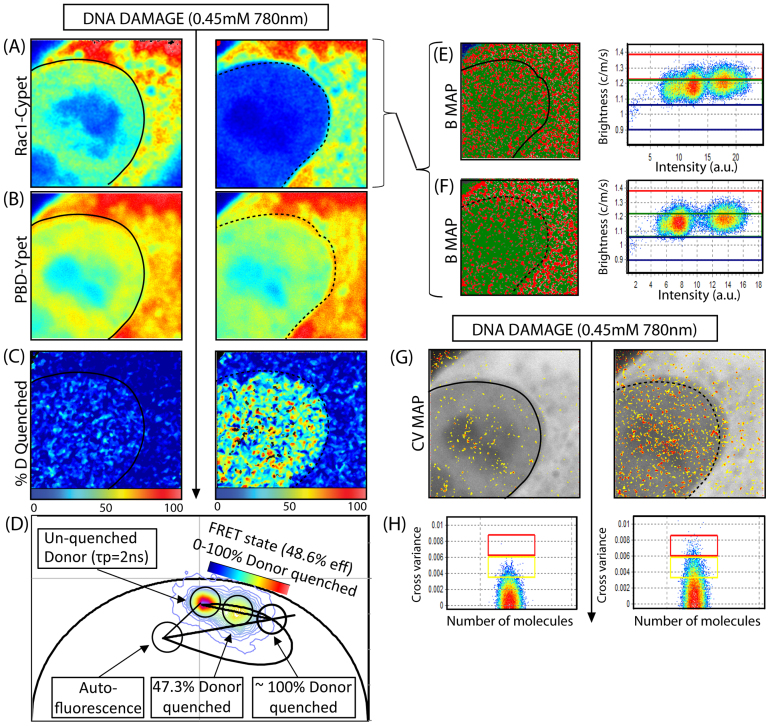
Wild type Rac1 FLARE biosensor activity and oligomerisation during the DNA damage response. (A)–(B) Intensity image of a NIH3T3 nucleus transiently transfected with Rac1-CyPet (donor) and PBD-YPet (acceptor) respectively, before (left) and immediately after (right) induction of DNA damage. Each FLIM/NB measurement takes 115 s to acquire and micro-irradiation takes 1.15 s. (C) FLIM images of the biosensor before (left) and after (right) DNA damage depicted in (A)-(B) and pseudo-colored according to the palette defined in (D), which spans 0–100% donor quenched from blue to red respectively. Note that after induction of DNA damage the nuclear population of Rac1 becomes active. (D) The phasor distribution of lifetimes measured for the Rac1 biosensor experiment depicted in (A)–(B), with the theoretical FRET trajectory superimposed to determine the percentage of donor molecules that are quenched between the un-quenched donor phasor blue (τ_phase_ = 2 ns) and the biosensor FRET state (τ_phase_ = 1 ns), which has a FRET efficiency of 48.6%. (E)–(F) Molecular brightness of Rac1-CyPet before (E) and after (F) DNA damage pseudo-coloured according to the coloured cursors superimposed over the brightness versus intensity distributions (right): monomers coloured green and dimers coloured red. Dimers are exported from the nucleus upon induction of DNA damage. (G) Cross variance analysis of biosensor FRET experiment depicted in (A)–(B) pseudo-colored according to the palette defined in (H). (H) Cross variance analysis of Rac1-CyPet intensity fluctuation with PBD-YPet intensity fluctuation recorded in each pixel of the image. In (A–C), (F) and (G), nuclear shape before and after DNA damage are indicated by solid and dashed black lines, respectively.

Using this multiplexed method of analysis, we assess the effect DNA damage has on Rac1 signalling and oligomerisation by acquisition of the fluorescence lifetime (phasor) and intensity in two channels of the dual chain Rac1 sensor before and after micro-irradiation of a NIH3T3 nucleus with a pulsed infrared 2-photon laser. The 2-photon laser was set to a power predetermined to recruit DNA repair factor NEIL2 DNA glycosylase (Figure S1)^19^. As can be seen in Figure 2A, prior to DNA damage Rac1-CyPet is accumulated in the nucleus (a cell cycle dependent localisation) and upon micro-irradiation, this accumulation dramatically changes to an exclusion of Rac1 from the nucleus (observed in N = 7 cells). In contrast, there is no significant change in the localisation of PBD-YPet (the binding partner of Rac1-CyPet, Figure 2B) upon induction of DNA damage, which suggests that Rac1-CyPet transport to the cytoplasm is not dependent on PBD binding. To assess Rac1 activity in terms of Rac1-CyPet-PBD-YPet FRET, we extracted FLIM data from the donor channel (as shown in Fig. 1D) to determine the spatial distribution of FRET. We pseudo-coloured the FLIM images (Figure 2C) according to the percentage of donor quenched from τ_phase_ = 2 ns (~0% quenched Rac1-CyPet) to τ_phase_ = 1.0 ns (~100% quenched Rac1-CyPet) (Figure 2D). The un-quenched and quenched lifetime of Rac1-CyPet, which we attribute to the FRET state of the biosensor (48.6% FRET efficiency), was verified by measuring the phasor position of the dominant negative (T17N) and constitutively active (Q61L) mutants of the Rac1 donor chain, respectively (Figure S2). We find from inspection of the FLIM images in Figure 2C that prior to DNA damage, Rac1 is predominantly inactive in both the nucleus and cytoplasm. Then immediately after micro-irradiation, there is a nuclear wide activation of Rac1 (Figure 2C) where 50.3 ± 13.9% of the donor molecules are quenched (p = 8.8 × 10^−6^, N = 10 cells despite 3/10 cells tested not having nuclear accumulation of Rac1 before induction of damage, due to the cell cycle dependence of Rac1 nuclear localisation (Figure S3))^3^. Induction of this FRET state by micro-irradiation with the 2-photon laser was specific to induction of DNA damage and not a general feature of 2-photon excitation since micro-irradiation of the cytoplasm under the same conditions did not induce Rac1 activation (Figure S4).

To assess whether the detected nuclear wide activation of Rac1 is regulated by the oligomeric state of the individual biosensor chains, we then carried out a moment analysis of the recorded intensity fluctuation in each pixel of the images (Figure 2A–B), to extract the molecular brightness (ε) of Rac1-CyPet or PBD-YPet before and after DNA damage. The measured apparent brightness (B_apparent_ = ε + 1) of monomeric Rac1-CyPet was determined as B_apparent_ = 1.15 (Figure S5) and given that this value represents the superimposition of the molecular brightness of background (B_background_ = 1) and the molecular brightness of CyPet (B_monomer_ = 0.15), we can extrapolate that a Rac1-CyPet dimer would have an apparent brightness of 1.30 (given that B_dimer_ = (2 × 0.15) = 0.3). Based on this prediction and the measured values, we found that Rac1-CyPet existed as a monomer (B_monomer_ = 1.15) and dimer (B_dimer_ = 1.30) throughout the cell before induction of DNA damage (Figure 2E). Then immediately after induction of DNA damage (Figure 2F), 43.5 ± 18.3% of the nuclear Rac1 dimer population was exported to the cytoplasm (p = 0.0025, N = 5 cells). This result, could in theory be an artefact of the high degree of FRET observed in the nucleus, given that the lifetime change associated with Rac1-CyPet undergoing FRET in the nucleus would result in a proportional decrease in molecular brightness of nuclear pixels. However, if we analyse the brightness of PBD-YPet from the same experiment before and after DNA damage (Figure S6), we do not find a proportional increase in brightness of the PBD-Ypet molecules in the nucleus upon induction of DNA damage, as would be expected if the redistribution of brightness detected in the donor channel is due to FRET interaction. Instead, we find PBD-YPet to be monomeric throughout the cell before and also after DNA damage. In addition, if we analyse the brightness of Rac1-CyPet in the absence of the PBD-YPet before and after DNA damage (Figure S7), we observe a dissociation of the dimeric population of Rac1-CyPet in the nucleus upon micro-irradiation, despite the fact that the acceptor and therefore any FRET interaction is not present in this experiment. Thus based on these two control experiments, the data presented in Figure 2E–F strongly suggest that the detected change in brightness of the nuclear pixels from 1.30 to 1.15 is correctly attributed to an export of Rac1 dimers upon micro-irradiation. A cross variance analysis of the Rac1-CyPet molecules with PBD-YPet before and after DNA damage (Fig. 2G-H) revealed association in 27.9 ± 4.2% more pixels upon induction of DNA damage (p = 6.18 × 10^−5^, N = 5 cells) and in agreement with the FLIM-FRET analysis, the pixels showing association (and therefore Rac1 activity) were localised in the nucleus (Fig. 2C). Thus fluctuation analysis is also sensitive to detection of Rac1 nuclear activation with the additional advantage of elucidating the oligomeric state of the individual biosensor chains (monomeric *versus* dimeric Rac1 by N&B) and their stoichiometry when complexed (Rac1-Cypet-PBD-YPet by cN&B). In conclusion, Rac1 in the nucleus is predominately inactive (Fig. 2C), existing as both dimer and monomer (Fig. 2E). Upon DNA damage, Rac1 is transported to the cytoplasm (Fig. 2A) where it accumulates as dimers (Fig. 2F) while the remaining nuclear monomeric Rac1 is activated (Fig. 2C) and complexes with PBD (Fig. 2G).

**Figure 3:**
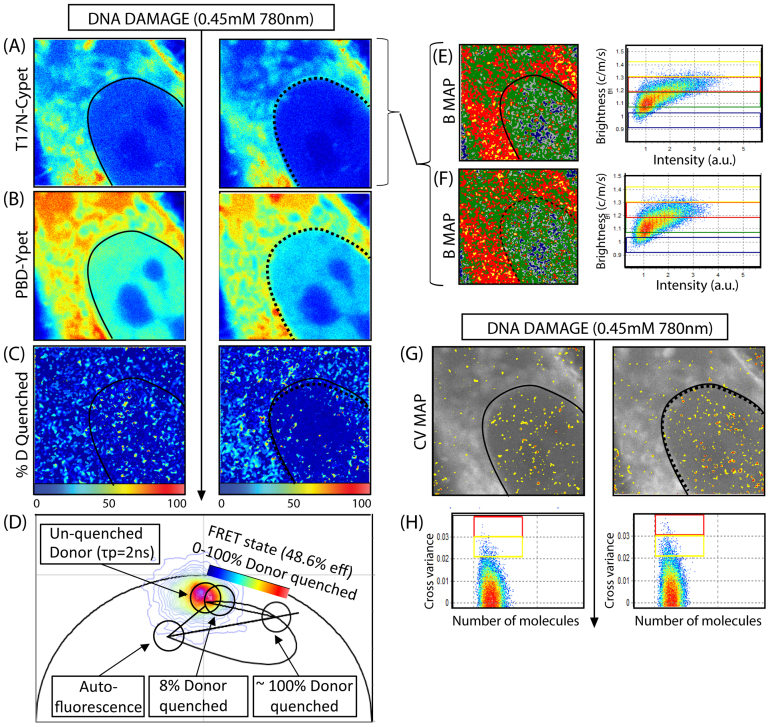
Inactive mutant of Rac1 (T17N) FRET biosensor activity and oligomerisation during the DNA damage response. (A)–(B) Intensity image of a NIH3T3 nucleus transiently transfected with T17N-CyPet (donor) and PBD-YPet (acceptor) respectively, before (left) and immediately after (right) induction of DNA damage. (C) FLIM images of the biosensor before (left) and after (right) DNA damage depicted in (A)–(B) and pseudo-colored according to the palette defined in (D), which spans the 0-100% blue to red range of donor quenching that is projected for the Rac1 biosensor FRET efficiency of 48.6%. As can be seen from this representation, after induction of DNA damage the inactive mutant T17N completely inhibited nuclear activation of Rac1 but resulted in activation of 8% of cytoplasmic Rac1. (D) The phasor distribution of lifetimes measured for the T17N biosensor experiment depicted in (A)–(B), with the theoretical FRET trajectory superimposed with respect to the unquenched donor (tau phase 2 ns) and background (auto-fluorescence). (E)–(F) Molecular brightness of T17N-CyPet before (E) and after (F) DNA damage pseudo-coloured according to the coloured cursors superimposed over the brightness versus intensity distributions (right): monomers coloured green, dimers coloured red, higher order oligomers coloured yellow. Dimers are excluded from the nucleus and this localisation does not change upon induction of DNA damage. (G) Cross variance analysis of biosensor FRET experiment depicted in (A)–(B) pseudo-colored according to the palette defined in (H). (H) Cross variance analysis of T17N-CyPet intensity fluctuation with PBD-YPet intensity fluctuation recorded in each pixel of the image. In (A–C), (F) and (G), nuclear shape before and after DNA damage are indicated by solid and dashed black lines, respectively.

To test whether Rac1 oligomerisation and translocation from the nucleus to the cytoplasm upon DNA damage depends on Rac1 GTPase activity, we expressed a dominant inactive mutant of Rac1 (T17N-Cypet) in the presence of PBD-Ypet and repeated the concomitant phasor and cross variance analysis. We found that T17N-Cypet was excluded from the nucleus prior to DNA damage in the majority of cells (5/8 cells) and this localisation was maintained upon exposure to infrared radiation (Fig. 3A–B). The lifetime analysis on the donor channel before and after DNA damage revealed that the Rac1 mutant was predominantly inactive in the nucleus before and after DNA damage with 12.7 ± 6.6% of donor quenched in the cytoplasm (p = 0.029, N = 8 cells despite 3/8 cells tested having a nuclear accumulation of Rac1 before induction of damage due to the cell cycle dependent nuclear localisation of Rac1 (Figure S8))^3^. The brightness analysis of the individual biosensor chains revealed that the inactive mutant of Rac1 existed in a dimeric and higher order oligomeric form (Figure 3E–F) in patches of the cytoplasm before and after DNA damage, and the small nuclear population of oligomer detected does not redistribute significantly (7.3 ± 2.9%) upon micro-irradiation (p = 0.418, N = 5 cells). Cross variance analysis before and after DNA damage (Fig. 3G–H) confirms the lack of nuclear association between T17N-CyPet and PBD-Ypet that was found by FLIM-FRET with only a 1.7 ± 0.9% increase in the number of pixels showing association and their localisation being throughout the entire cell (p = 0.303, N = 5 cells). Thus in conclusion it appears that Rac1 oligomerisation can occur even though T17N-CyPet does not interact with PBD-YPet, however, Rac1 activity is crucial for the change in nuclear localisation of Rac1 and redistribution of its oligomeric forms upon induction of DNA damage that was observed for wild type Rac1 in Figure 2.

### Cross number and brightness analysis of Rac1 interaction during nucleo-cytoplasmic transport

Our data so far indicate that DNA damage causes a re-distribution and spatial separation of active *versus* inactive Rac1 between the nucleus and cytoplasm, respectively and that this re-distribution and activation is related to oligomeric state, with the dimeric and inactive populations of Rac1 being selectively exported to the cytoplasm while nuclear Rac1 is active and monomeric. If we analyse the diffusive behaviour of the inactive Rac1-CyPet population from Figure 2 however (Figure S9), we find that although the population of Rac1-Cypet exported to the cytoplasm is inactive (as judged by an absence of FRET interaction with PBD-Ypet) this population is markedly slowed down (6.3 ± 2.9 μm^2^/s) as compared to the rate at which it diffused before DNA damage (11.2 ± 3.9 μm^2^/s) (N = 5 cells). Thus Rac1 appears to participate in a DNA damage-induced cytoplasmic binding events that do not require Rac1 activation.

**Figure 4:**
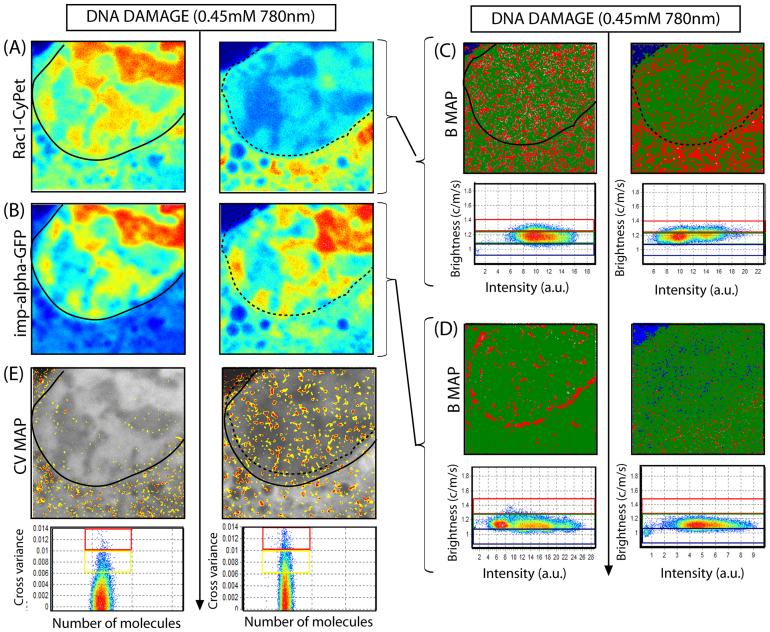
Wild type Rac1 interaction with importin-α2 during the DNA damage response. (A)–(B) Intensity image of a NIH3T3 nucleus transiently transfected with Rac1-CyPet importin-α-GFP respectively, before (left) and immediately after (right) induction of DNA damage. (C) Molecular brightness of Rac1-CyPet before (left) and after (right) DNA damage pseudo-coloured according to the coloured cursors superimposed over the brightness versus intensity distributions: monomers coloured green and dimers coloured red. Dimers are exported from the nucleus upon induction of DNA damage. (D) Molecular brightness of importin-α-GFP before (left) and after (right) DNA damage pseudo-coloured according to the coloured cursors superimposed over the brightness versus intensity distributions: monomers coloured green and dimers coloured red. Dimeric population along the nuclear envelope is disassembled upon induction of DNA damage. (E) Cross variance analysis of Rac1-CyPet with importin-α-GFP in each pixel of the image, before (left) and after (right) DNA damage. A nuclear association of Rac1-CyPet with importin-α-GFP is induced in addition to a cytoplasmic association, upon induction of DNA damage.

Since the cN&B analysis is sensitive and capable of detection of Rac1-CyPet interactions in the nucleus and cytoplasm, we thus investigated the translocation of Rac1 in the context of other Rac1 binding partners. Biochemical studies demonstrated that nuclear import receptor importin-α (also known as karyopherin α) is a direct interaction partner of Rac1, although it has only been shown as necessary for Rac1 transport from the cytoplasm to the nucleus^20^. We thus probed this interaction using only cross Number and Brightness analysis since there is no FRET biosensor to test this association. We expressed Rac1-CyPet and importin-α-GFP in NIH3T3 cells. As expected, Rac1-CyPet was localised in the nucleus and cytoplasm and upon micro-irradiation was dramatically transported to the cytoplasm (Fig. 4A). Concomitantly, importin-α-GFP was predominately localised in the nucleus and upon micro-irradiation a significant portion of importin-α-GFP is transported to the cytoplasm (Fig. 4B, observed in N = 7 cells). In agreement with Fig. 2-E–F, the brightness analysis of Rac1 (Figure 4C) demonstrated that Rac1 existed as a monomer and dimer throughout the cell and upon DNA damage 33.1% of the nuclear population of dimeric Rac1 was transported to the cytoplasm. From calibration of monomeric EGFP's molecular brightness as ε = 0.2 (Figure S10), brightness analysis of importin-α-GFP (Fig. 4D) revealed that importin-α formed a dimeric population around the nuclear envelope that disappeared upon micro-irradiation (N = 7 cells). Thus at the same time that Rac1 and importin-α were transported to the cytoplasm, a dimeric population of importin-α along the nuclear envelope was disassembled. Cross variance analysis of Rac1-CyPet interaction with importin-α (Fig. 4E) indicates that although prior to DNA damage, these two proteins formed a complex that is limited to the cytoplasm (in agreement with the biochemical data^20^), micro-irradiation induced Rac1-CyPet association with importin-α-GFP in 21.9 ± 4.8% more pixels and the pixels showing induced association were largely localised in the nucleus (p = 0.0022, N = 5 cells). Thus importin-α-GFP appears to mediate not only the import of Rac1-Cypet to the nucleus but also the export.

**Figure 5:**
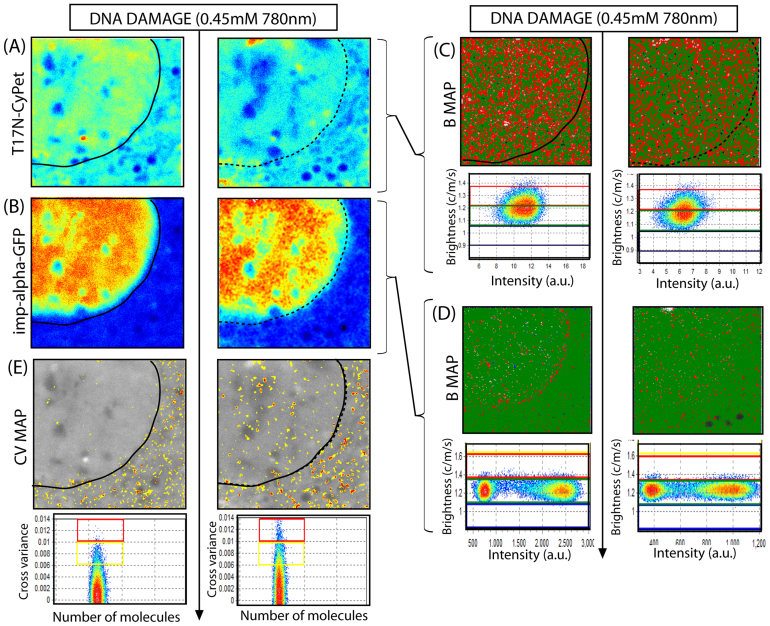
Inactive mutant of Rac1 (T17N) interaction with importin-α2 during the DNA damage response. (A)–(B) Intensity image of a NIH3T3 nucleus transiently transfected with T17N-CyPet and importin-α-GFP respectively, before (left) and immediately after (right) induction of DNA damage. (C) Molecular brightness of T17N-CyPet before (left) and after (right) DNA damage pseudo-coloured according to the coloured cursors superimposed over the brightness versus intensity distributions: monomers coloured green and dimers coloured red. Oligomeric distribution of T17N-CyPet is unchanged upon induction of DNA damage. (D) Molecular brightness of importin-α-GFP before (left) and after (right) DNA damage pseudo-coloured according to the coloured cursors superimposed over the brightness versus intensity distributions: monomers coloured green and dimers coloured red. Dimeric population along the nuclear envelope is suppressed in the presence of T17N. (E) Cross variance analysis of T17N-CyPet with importin-α-GFP in each pixel of the image, before (left) and after (right) DNA damage. Negligible nuclear association of T17N-CyPet with importin-α-GFP detected upon induction of DNA damage.

To test whether this association is dependent on Rac1 activity, we examined the relationship between T17N-CyPet and importin-α-GFP before and after DNA damage. As can be seen in Figure 5A, T17N-CyPet was highly localised in the selected nucleus and unlike wild-type Rac1, the nuclear population of T17N-CyPet was not exported to the cytoplasm upon DNA damage (Figure 5A). This strongly suggests that Rac1 activity is required for DNA-damage induced nuclear export of Rac1 while Rac1-PBD binding was not (Figure 2B). Examining the intracellular localisation of importin-α-GFP (Figure 5B) we found in agreement with Figure 4B, prior to DNA damage importin-α is only localised in the nucleus. However, upon micro-irradiation importin-α-GFP remained exclusively localised in the nucleus and was not transported to the cytoplasm when Rac1 activity was disabled. The brightness analysis of T17N (Figure 5C) shows that T17N formed a monomer and dimer throughout the entire cell, and in agreement with Figure 3E–F, induction of DNA damage did not significantly change this distribution of oligomer (4.5% reduction in dimer). Thus in the absence of Rac1 activity, oligomeric state does not regulate Rac1 localisation in response to DNA damage. The effect of Rac1 activity on importin-α can also be seen in the brightness analysis of importin-α-GFP before and after DNA damage (Figure 5D). When Rac1 was inactive, importin-α-GFP formation of dimer around the nuclear envelope was supressed in undamaged cells. In line with this result, the cross variance analysis of T17N-CyPet interaction with importin-α-GFP (Figure 5E) revealed that prior to DNA damage T17N-CyPet associated with importin-α-GFP in the cytoplasm (in agreement with the wild type experiment presented in Figure 4) but upon induction of DNA damage, the cross N&B analysis detected only a 3.5 ± 1.4% increase in the number of pixels showing association and the localisation of these pixels was throughout the entire cell (p = 0.216, observed in N = 5 cells). Thus our data indicate that inhibition of endogenous Rac1 activity with the dominant inactive Rac1 mutant (T17N) disrupts importin-α-GFP formation of dimer around the nuclear envelope and subsequent translocation of this nuclear import factor to the cytoplasm upon induction of DNA damage.

## Conclusion

Here we establish a method to assess how protein stoichiometry modulates signalling protein activity that is based on the acquisition of fluorescence lifetime imaging microscopy (FLIM) data in a format that enables concomitant phasor and Number and Brightness (N&B) analysis. We demonstrated this multiplexed approach on the Rac1 FRET biosensor detection and showed that this method not only enables measurement of the oligomeric state of the donor, acceptor and subsequent stoichiometry upon forming a complex, but also verifies the degree of signalling activity detected by FLIM-FRET through a cross variance analysis. We also demonstrated the ability of cross Number and Brightness (cN&B) alone to probe different binding partners without need for design of a FRET biosensor.

We used this multiplexed method to investigate the role Rac1 activation plays in the DNA damage response pathway. We found that upon induction of DNA damage, wild type Rac1 partitions itself between the nucleus and cytoplasm, placing inactive Rac1 molecules (Rac1-Cypet) in the cytoplasm and active Rac1 molecules (Rac1-CyPet-PBD-YPet) in the nucleus. This redistribution requires Rac1 activation and appears to be selective in terms of the oligomeric state of Rac1, given that only dimers of wild type Rac1 accumulated in the cytoplasm. We then investigate the mechanism by which Rac1 underwent this nucleo-cytoplasmic shuttling by probing interaction with importin-α-GFP (an identified potential binding partner). We found from brightness and cross variance analysis that Rac1 import/export is indeed mediated by an unusual relationship with this nuclear import receptor. In particular we noticed that prior to DNA damage, importin-α-GFP existed as a dimer around the nuclear envelope (N&B) and only associated with Rac1 in the cytoplasm (cN&B). Upon induction of DNA damage, the importin-α-GFP dimeric population dissociates and Rac1 association with importin-α-GFP was observed in the nucleus in addition to the cytoplasm. Perhaps most intriguingly, we found that importin-α-GFP not only regulates Rac1 distribution but also the reverse, that Rac1 affects importin-α-GFP organisation. That is inhibition of endogenous Rac1 with the inactive mutant of Rac1 (T17N) inhibited translocation of this nuclear import factor to the cytoplasm and diminished importin-α-GFP formation of dimer around the nuclear envelope upon induction of DNA damage.

In conclusion, our data suggest not only that Rac1 signalling has a direct role in the regulation of nucleo-cytoplasmic transport during DNA repair but also demonstrate the influence of Rac1 signalling on nuclear organisation. Only with this multiplexed approach, which visualises both signalling protein activity and oligomerisation, could we have made this novel observation, which potentially has widespread effects on nucleo-cytoplasmic transport in general during DNA damage repair.

## Methods

### Cell culture

NIH3T3 cells were grown in high glucose medium from Invitrogen, supplemented with10% Fetal Bovine Serum, 5 ml of Pen-Strep and HEPES at 37°C and in 5% CO_2_. Freshly split cells were plated onto 35-mm glass bottom dishes coated with fibronectin and then, after twenty four hours, transiently transfected with either the wild type, inactive or constitutively mutant of the Rac1 FLARE dual chain FRET biosensor sourced from the Hahn's Lab, University of North Carolina 27599, USA. The chains of these three Rac1 biosensors are: (1) Rac1 wild type dual chain biosensor (CyPet-Rac1 and YPet-PBD) (2) Rac1 inactive mutant dual chain biosensor (CyPet-T17N and YPet-PBD) (3) Rac1 constitutively-active mutant dual chain biosensor (CyPet-Q61L and YPet-PBD). For the karyopherin-alpha-2 experiments cells were transiently co-transfected with imp-alpha tagged to EGFP. Details about the importin-α-GFP plasmid can be found in reference^21^. In either case the plated cells were then left for a further twenty four hours at 37°C/5% CO_2_.

### Induction of dna damage by micro-irradiation

For all micro-irradiation experiments the 2-photon Ti:Sapphire laser (80 fs, repetition rate 80 MHz, Spectra-Physics Mai Tai, Newport Beach) was tuned to 780 nm and used in conjunction with the Zeiss LSM710 META laser scanning microscope^19^. The laser beam was then focused on a small section of the nucleus (5 μm × 5 μm) which avoided the nucleolus or nuclear envelope and a frame scan acquired (256 × 256 pixels, 21 μs/pixel, total acquisition time 1.15 s) at a power level of 0.5 mW, as measured at the objective. These conditions were found to recruit DNA repair factor NEIL2 (Figure S1).

### Microscope

For the FRET biosensor experiments FLIM and Number and Brightness data were acquired concomitantly with the Zeiss LSM710 META laser scanning microscope, coupled to a 2-photon Ti:Sapphire laser (Spectra-Physics Mai Tai, Newport Beach) producing 80 fs pulses at a repetition of 80 MHz, and a ISS A320 FastFLIM box to acquire the lifetime data in the digital frequency domain^22^. A 40× water immersion objective 1.2 N.A. (Zeiss, Germany) was used for all experiments. The 2-photon excitation laser was tuned to 900 nm for excitation of the donor and acceptor fluorophores, as this wavelength was found to not cause DNA damage. A SP 760 nm dichroic filter was used to separate the fluorescence signal from the laser light. The fluorescence signal was directed through a 509 LP CFP/YFP filter, and the donor and acceptor signal split between two photomultiplier detectors (H7422P-40 of Hamamatsu), with the following bandwidth filters in front of each: CFP 470/22 and YFP 542/27, respectively.

### Image acquisition

For image acquisition the pixel frame size was set to 256 × 256 at an electronic zoom that resulted in a pixel size of 50–100 nm (image size of 12.8 μm). This spatial scale was selected to enable concomitant RICS analysis, a spatiotemporal correlation function which requires oversampling of a diffusing molecule in space to determine the rate of motion in time. A stack of 100 frames was then acquired at this zoom with the pixel dwell time set to 25.61 μs/pixel, which resulted in a line time of 6.24 ms, a frame time of 1.15 s and an overall acquisition time of 115 s. This temporal scale was selected to (1) ensure sufficient counts were integrated per pixel for phasor calculation (lifetime analysis) and (2) enable a scan rate comparable to the rate of Rac1 diffusion so that fluctuation analysis of Rac1 molecular brightness (N&B) and diffusion (RICS) could be performed. The average laser power at the sample was maintained at the 1–2 mW level. The intensity and time resolved data measured for FLIM, Number and Brightness and RICS analysis, were acquired and processed by the SimFCS software developed at the Laboratory for Fluorescence Dynamics (www.lfd.uci.edu). Calibration of the phasor plot space for FLIM analysis was performed by measuring fluorescein (pH 9.0), which has a known single exponential lifetime of 4.04 ns. Calibration of the monomeric brightness of each fluorescent construct for N&B analysis was performed by measurement of cells transfected with free CyPet, YPet or EGFP under identical experimental conditions (Figure S5, S6 and S10). Calibration of the point spread function (PSF) for RICS analysis was performed by measurement of EGFP diffusion (90 μm^2^s^−1^) and found to be approximately 260 nm.

### FLIM-FRET data analysis

The phasor transformation and data analysis were performed using the SimFCS software available at the www.lfd.uci.edu, as described in previously published papers^14^,^16^. Briefly, the fluorescence decay in each pixel of the FLIM line scan is transformed into the sine and cosine components which are then represented in a two dimensional polar plot (phasor plot), according to equations 1 and 2: 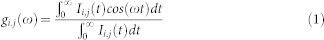

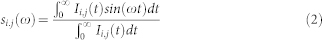
where ω is the laser repetition angular frequency (2πf) and the indexes *i* and *j* identify a pixel of the image. Each pixel of the FLIM image thus gives rise to a single point (phasor) in the phasor plot and when used in reciprocal mode, enables each point of the phasor plot to be mapped to each pixel of the FLIM image. Since phasors follow simple vector algebra, it is possible to determine the fractional contribution of two or more independent molecular species coexisting in the same pixel. Thus in the case of two independent species, all possible weightings give a phasor distribution along a linear trajectory that joins the phasors of the individual species in pure form^16^.

In the case of a FRET experiment where the lifetime of the donor molecule is changed upon interaction with an acceptor molecule, the realization of all possible phasors quenched with different efficiencies describe a curved trajectory in the phasor plot^14^. The experimental position of the phasor of a given pixel along the trajectory determines the amount of quenching and therefore the FRET efficiency of that location. The contributions of the background and of the donor without acceptor are evaluated using the rule of the linear combination, with the background phasor and the donor unquenched determined independently. In the case of the dual chain biosensor which has a fixed FRET efficiency, the linear combination of phasor clusters between the donor phasor and FRET state of the biosensor represent the varying contributions of donor fluorescence and quenched donor fluorescence in any one pixel. By moving the phasor cursor along the straight line drawn between these two terminal phasor locations, we can calculate the exact fractional contribution of quenched donor fluorescence for each pixel highlighted^14^. We use this fractional analysis to determine the percentage of donor molecules quenched in the nucleus before and after DNA damage for each cell tested. We then average the percentage determined for this region of interest for n cells, quote the standard deviation of this recorded distribution and perform a Student T test (2-tail) to compare the percentage of donor molecules quenched before DNA damage with the percentage quenched after DNA damage.

### Number and brightness (N&B) and cross variance data analysis

Calculation of the Number and Brightness or cross variance of molecules in each pixel of an image was performed using the SimFCS software available at www.lfd.uci.edu, as described in previously published papers^17^,^18^. Briefly in each pixel of a time series of frames we have an intensity fluctuation that has an average intensity 〈k〉 (first moment) and a variance σ^2^ (second moment). These two properties describe the apparent number (N) and brightness (B) of the molecules that give rise to the intensity fluctuation, according to equations 3 and 4: 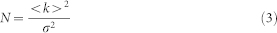

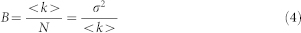


The true molecular brightness (ε) of the molecules that give rise to the measured apparent brightness (B) in the case of a photon counting detector are related according to equation 5: 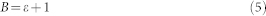
where 1 is the brightness contribution of the detector given that the photon counting detector variance (σ^2^_detector_) should equal the average intensity of the detector noise (〈k〉_detector_). In the case of an analogue detector (as was used in the results presented here) this is not true due to characteristics of the analog amplifier and the settings of the analog-to-digital converter. Thus the detector's brightness contribution needs to be accounted for by a term we call the S factor, which returns the background brightness to 1 so that the molecular brightness of the molecules can be extracted. By analysis of pixels that did not contain fluorescence from the sample, we found the brightness of the background to be between 1.5–1.9 depending on the laser power required. Taking into account this S factor (1.5–1.9) we then calibrated the monomeric brightness of free CyPet, YPet and EGFP, so that in the instance oligomerisation was observed when any one of these constructs was tagged to the different proteins of interest we could extract the stoichiometry of the oligomer (as described in Figure S5, S6 and S10 respectively). The percentage of Rac1 dimers exported from the nuclei of the different cells tested was calculated from the difference in the number of pixels which gave rise to a dimeric brightness in this region of interest, before and after DNA damage. The cross variance in each pixel of a frame acquisition was calculated according to equation 6: 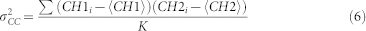
The fraction of pixels which gave rise to a positive cross variance above what is symmetrical below the zero level, was quantified for Rac1-CyPet or T17N-CyPet interaction with PBD-YPet or importin-α-GFP before and then after DNA damage. The change induced was then expressed as a percentage increase of the number of pixels with association upon induction of DNA damage in each cell tested. For both the change in the percentage of dimer detected in the nucleus and the percentage of pixels showing cross variance, a Student T test (2-tail) was performed to compare these values before and after DNA damage.

### Raster image correlation spectroscopy (RICS) data analysis

Calculation of the RICS and cross RICS function to derive the diffusion coefficient of Rac1-CyPet as well as the degree of association with PBD-YPet or importin-alpha in each pixel of an image was performed using the SimFCS software available at www.lfd.uci.edu, as described in previously published papers^23^,^24^,^25^,^26^.

## Author Contributions

E.H. and E.G. designed research; E.H. performed research; K.M.H., K.Y. and K.G. contributed new reagents/analytic tools; E.H. and E.G. analyzed data; and E.H. wrote the paper. All authors reviewed the manuscript.

## Supplementary Material

Supplementary Information Supplementary Material
