# Supplementary material for: Fluctuation-based imaging of nuclear Rac1 activation by protein oligomerisation
Source: Sci Rep. 2014 Feb 27;4:4219. doi: 10.1038/srep04219 (PMC3936235; doi:10.1038/srep04219)
Supplement: Supplementary Information — Supplementary Material [file srep04219-s1.pdf]

SUPPLEMENTARY INFORMATION

Fluctuation-based imaging of nuclear Rac1 activation by protein oligomerization

Elizabeth Hinde[a,c*], Kyoko Yokomori[b], Katharina Gaus[c], Klaus Hahn[d], Enrico Gratton[a]

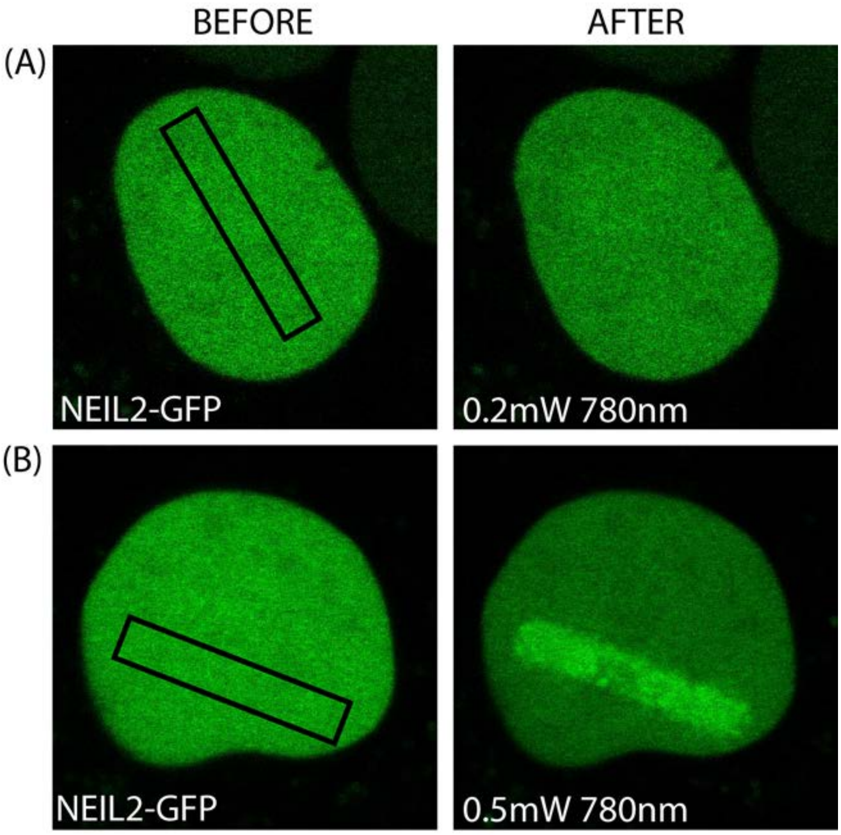

FIGURE S1: Optimisation of micro-irradiation conditions for DNA repair factor recruitment with a 2-photon laser. (A) Micro-irradiation of a NIH3T3 nucleus with the Ti Sapphire 2-Photon laser operated at 780nm, 0.2mW for 1.15s does not recruit DNA repair factor NEIL2. (B) Micro-irradiation of a NIH3T3 nucleus with the Ti Sapphire 2-Photon laser operated at 780nm, 0.5mW for 1.15s does recruit DNA repair factor NEIL2.

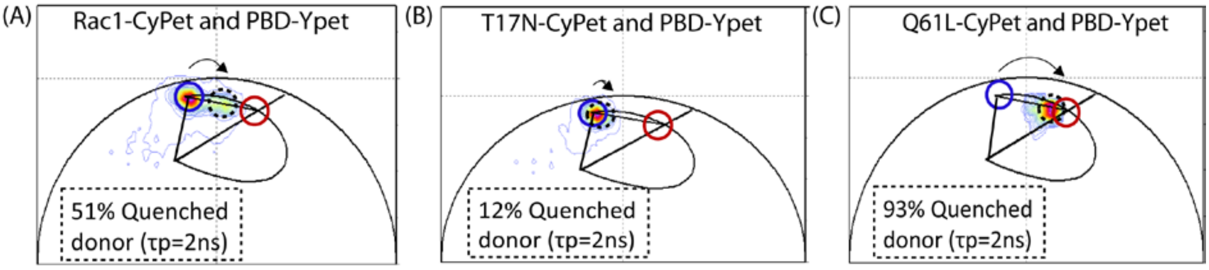

FIGURE S2: FLIM analysis of the inactive (T17N) and constitutively active (Q61L) mutants of Rac1 to confirm the phasor positions of the un-quenched donor and FRET state of the wild type Rac1 dual chain FRET biosensor (respectively). (A) Wild type FRET experiment (Rac1-CyPet and PBD-YPet) shows a shift in the Rac1-CyPet tau phase lifetime from 2ns toward 1.65ns upon induction of DNA damage. We quantify this shift to be the result of 51% of the donor molecules being quenched, given that this phasor distribution is a linear combination of the phasor clusters between: (1) the donor phasor (~0% quenched donor), as confirmed by the phasor position of the dominant negative mutant in (B) and (2) the FRET state (~100% quenched donor), as confirmed by the phasor position of the constitutively active mutant in (C).

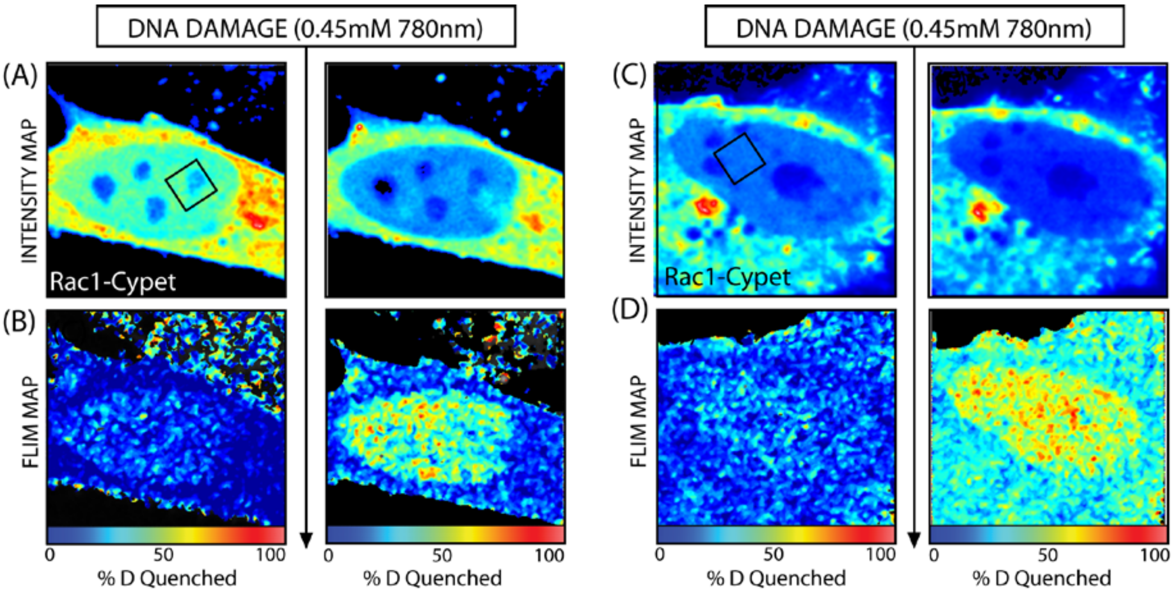

FIGURE S3: Nuclear localisation of Rac1 (accumulation or exclusion) does not affect nuclear wide activation of Rac1 upon induction of DNA damage. (A) Intensity image of a NIH3T3 nucleus transiently transfected with Rac1-CyPet (donor) and PBD-YPet (acceptor) respectively, before (left) and after (right) induction of DNA damage. Initial nuclear localisation of Rac1 shows accumulation. (B) FLIM images of the biosensor before (left) and after (right) DNA damage depicted in (A) and pseudo-coloured according to Rac1 activity (blue is low activity, red is high activity). After induction of DNA damage the nuclear population of Rac1 becomes active. (C) Intensity image of a NIH3T3 nucleus transiently transfected with Rac1-CyPet (donor) and PBD-YPet (acceptor) respectively, before (left) and after (right) induction of DNA damage. Initial nuclear localisation of Rac1 shows exclusion. (D) FLIM images of the biosensor before (left) and after (right) DNA damage depicted in (C) and pseudo-colored according to Rac1 activity (blue is low activity, red is high activity). Thus similarly to the experiment presented in (A)-(B) after induction of DNA damage the nuclear population of Rac1 becomes active.

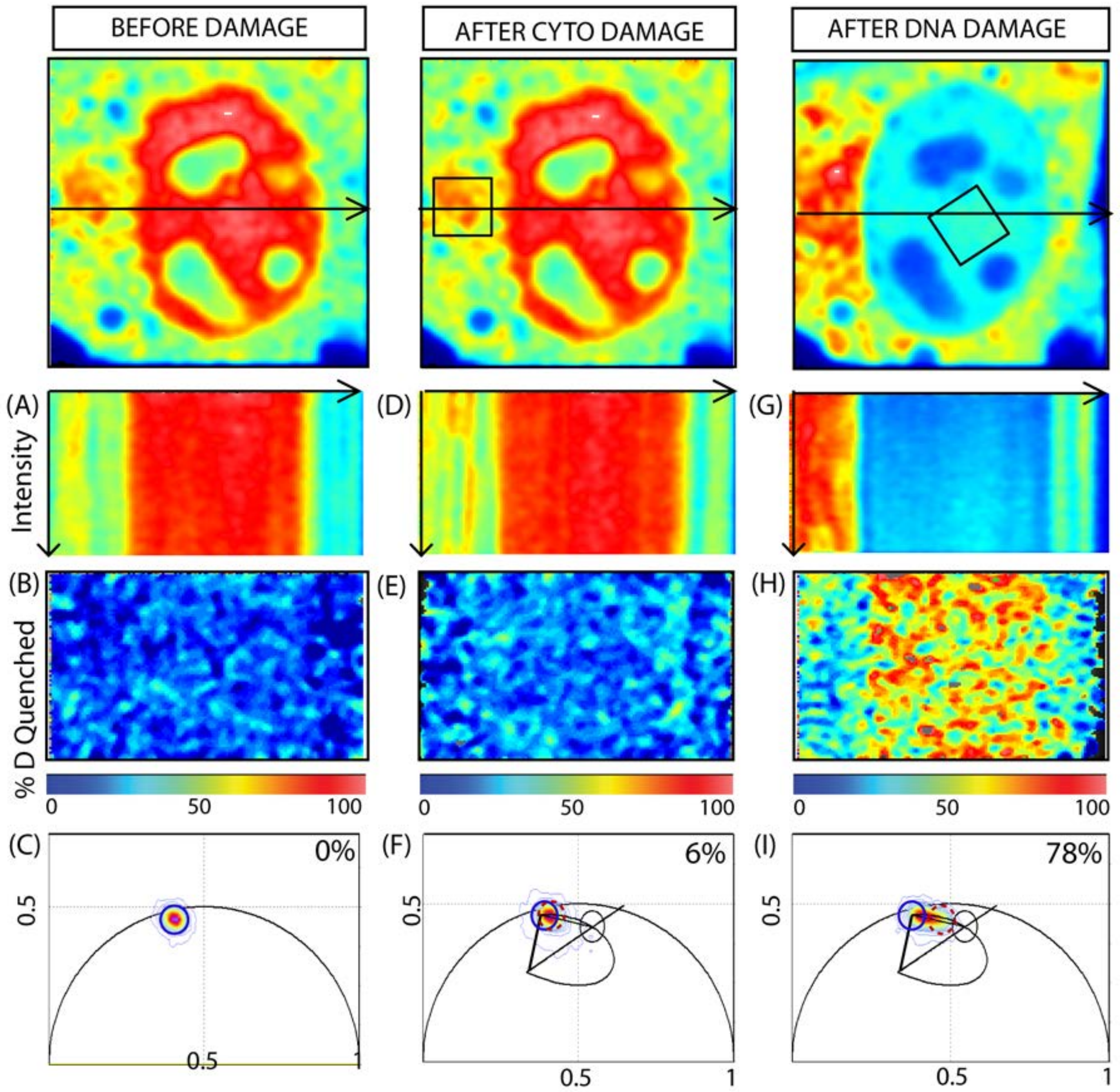

FIGURE S4: Micro-irradiation of the NIH3T3 cytoplasm confirms that the detected nucleus-wide activation of Rac1 is not an artefact of 2-photon excitation and a specific response to induction of DNA damage. (A)-(C) Intensity image of an NIH3T3 nucleus expressing Rac1-CyPet and line acquired across (A) to derive a FLIM line scan of the donor chain in the presence of PBD-YPet (B) pseudo-coloured according to the palette defined in the phasor plot (C). As can be seen Rac1-CyPet is un-quenched and exists as a single distribution characteristic of the un-quenched phasor position. (D)-(F) Intensity image of the NIH3T3 nucleus expressing Rac1-CyPet showing where micro-irradiation was induced in the cytoplasm and the line acquired across (D) to derive the FLIM line scan of the donor chain in the presence of PBD-YPet (E) pseudo-coloured according to the palette defined in the phasor plot (F). As can be seen 2-photon excitation of the cytoplasm induces a negligible shift in phasor of Rac1-CyPet and only 6% of the molecules are quenched. (G)-(I) Intensity image of the NIH3T3 nucleus expressing Rac1-CyPet showing where micro-irradiation was induced in the nucleus and the line acquire across (G) to derive the FLIM line scan of this donor chain in the presence of PBD-YPet (H) pseudo-coloured according to the palette defined in the phasor plot (I). As can be seen 2-photon excitation of the nucleus induces a nuclear wide activation of Rac1, as indicated by 78% of the molecules becoming quenched.

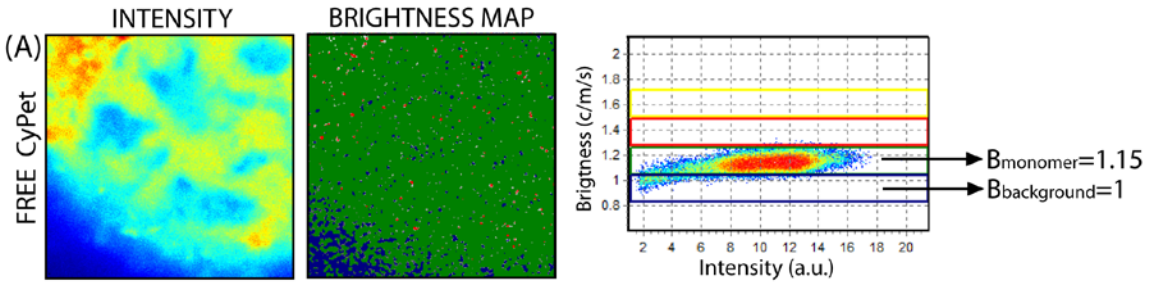

FIGURE S5: Calibration of the monomeric brightness of CyPet to enable extrapolation of the expected apparent brightness of Rac1-CyPet oligomers. (A) Brightness analysis of free CyPet within the nucleus and cytoplasm of an NIH3T3 cell reveals the monomeric distribution of this fluorescent protein to be centred at B=1.15, after correction for the analogue detector (brightness of the background returned to B=1). Thus the molecular brightness of CyPet is 0.15 and we can extrapolate that the apparent brightness of a Rac1-CyPet monomer would be (1+1*0.15)=1.15, a Rac1-CyPet dimer would be (1+0.15*2)=1.3, a Rac1-CyPet trimer would be (1+0.15*3)=1.45 and so on.

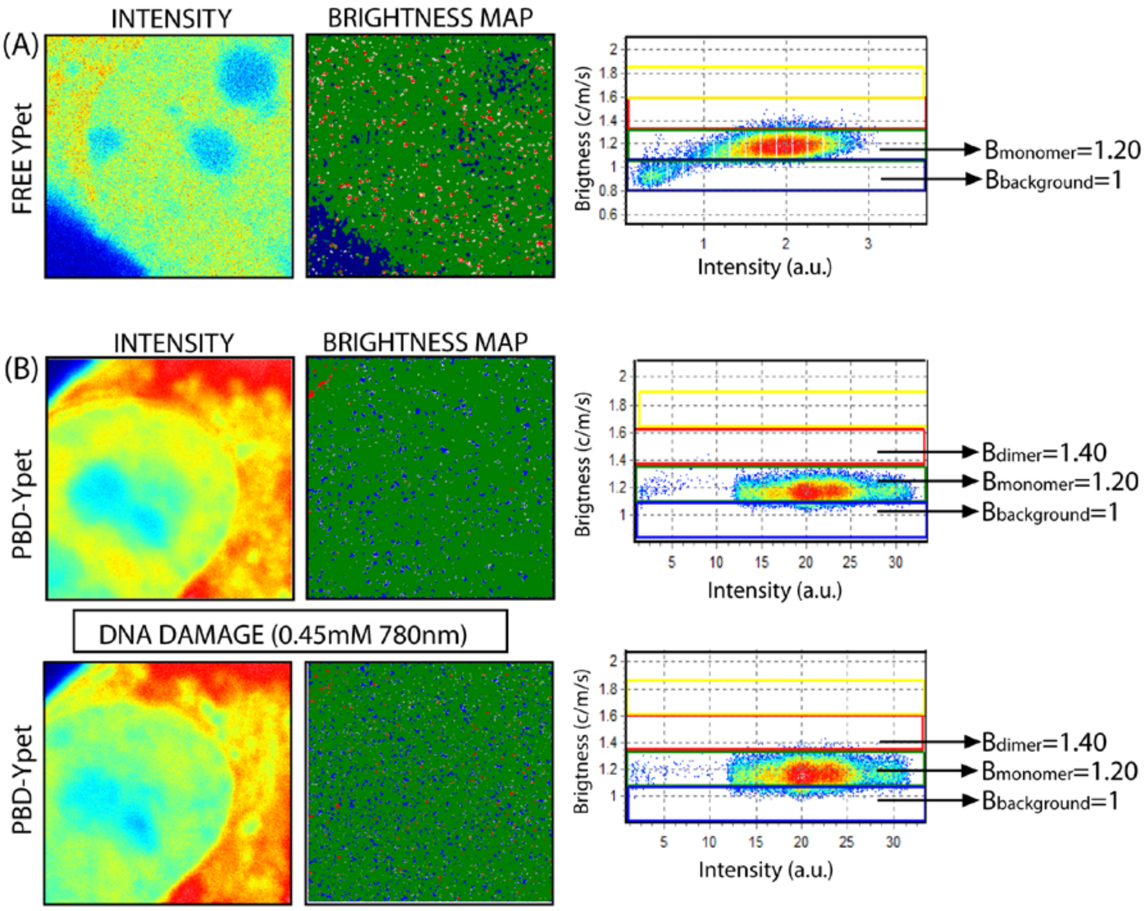

FIGURE S6: Brightness analysis of PBD-Ypet (data from experiment presented in Figure 2) reveals this construct to be monomeric and unchanged in oligomeric state upon induction of DNA damage. (A) Brightness analysis of free YPet within the nucleus and cytoplasm of an NIH3T3 cell reveals the monomeric distribution of this fluorescent protein to be centred at B=1.2, after correction for the analogue detector (brightness of the background returned to B=1). Thus the molecular brightness of YPet is 0.2 and we can extrapolate that the apparent brightness of a PBD-YPet monomer would be (1+1*0.2)=1.2, a PBD-YPet dimer would be (1+0.2*2)=1.4, a PBD-YPet trimer would be (1+0.20*3)=1.6 and so on. (B) Brightness analysis of PBD-YPet, from the experiment presented in Figure 2, reveals this construct to be monomeric throughout the cell and this oligomeric state is unchanged upon induction of DNA damage.

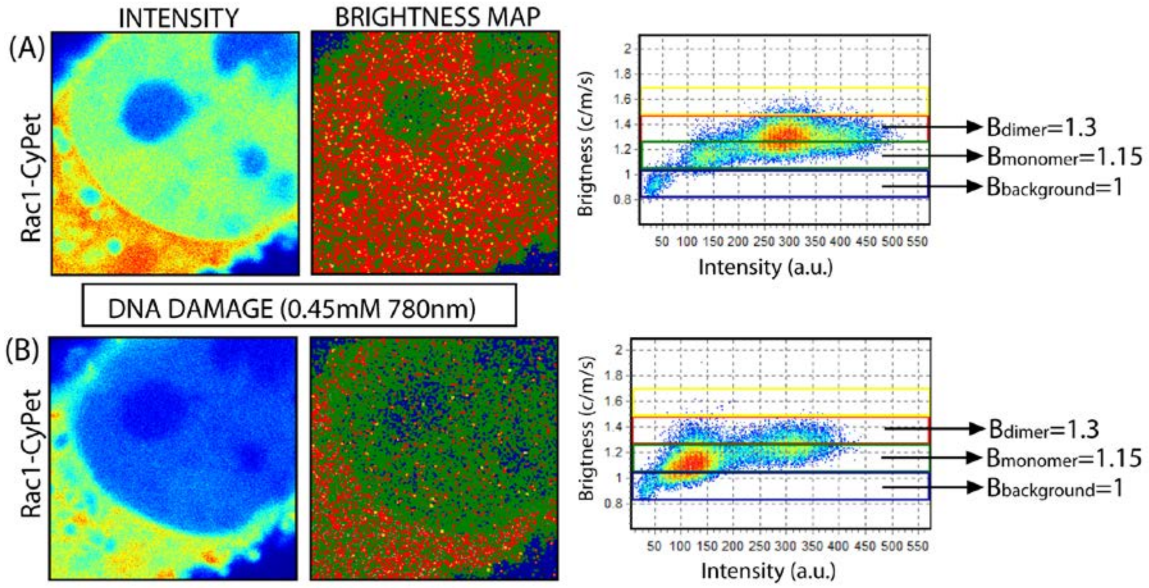

FIGURE S7: Brightness analysis of Rac1-CyPet in the absence of PBD-YPet reveals an analogous change in Rac1 oligomeric distribution to experiments conducted in the presence of PBD-YPet upon induction of DNA damage. (A) Before DNA damage Rac1-CyPet is monomeric and dimeric throughout the nucleus and cytoplasm. (B) Then upon induction of DNA damage, in agreement with Figure 2 we find the Rac1-CyPet dimers to be exported to the cytoplasm and the nuclear pool of Rac1-CyPet that remains is monomeric.

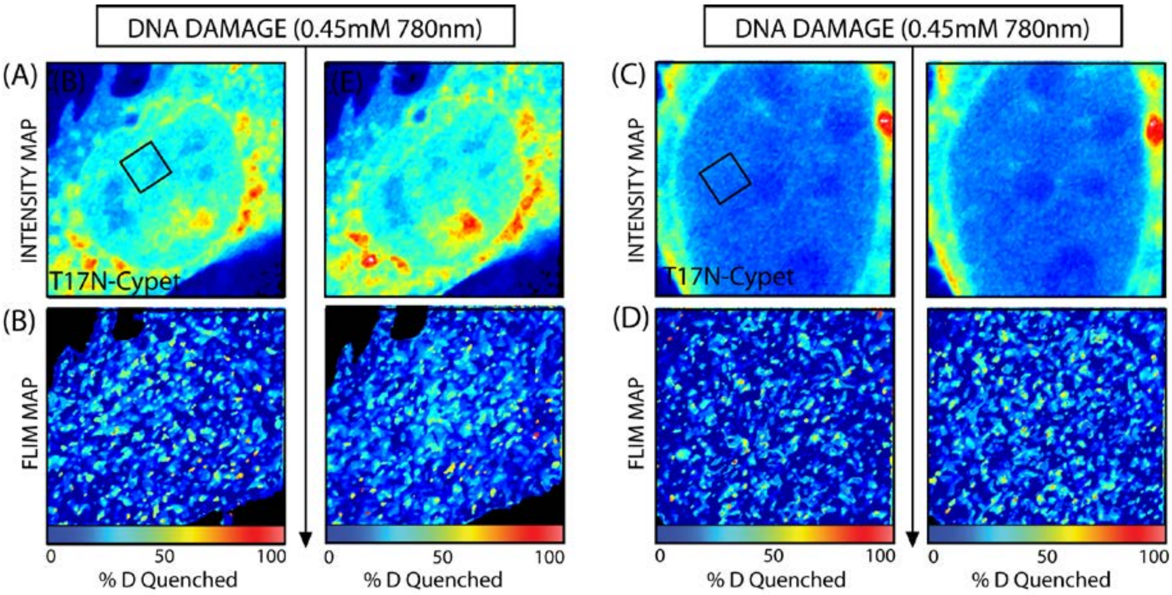

FIGURE S8: Nuclear localisation of T17N (accumulation or exclusion) does not affect the activation of profile of Rac1 detected upon induction of DNA damage. (A) Intensity image of a NIH3T3 nucleus transiently transfected with T17N-CyPet (donor) and PBD-YPet (acceptor) respectively, before (left) and after (right) induction of DNA damage. Initial nuclear localisation of Rac1 shows accumulation and remains unchanged after DNA damage. (B) FLIM images of the biosensor before (left) and after (right) DNA damage depicted in (A) and pseudo-coloured according to Rac1 activity (blue is low activity, red is high activity). After induction of DNA damage the nuclear population of T17N remains inactive. (C) Intensity image of a NIH3T3 nucleus transiently transfected with T17N-CyPet (donor) and PBD-YPet (acceptor) respectively, before (left) and after (right) induction of DNA damage. Initial nuclear localisation of Rac1 shows exclusion and this remains unchanged after DNA damage. (D) FLIM images of the biosensor before (left) and after (right) DNA damage depicted in (C) and pseudo-colored according to Rac1 activity (blue is low activity, red is high activity). Thus similarly to the experiment presented in (A)-(B) after induction of DNA damage the nuclear population of Rac1 remains inactive.

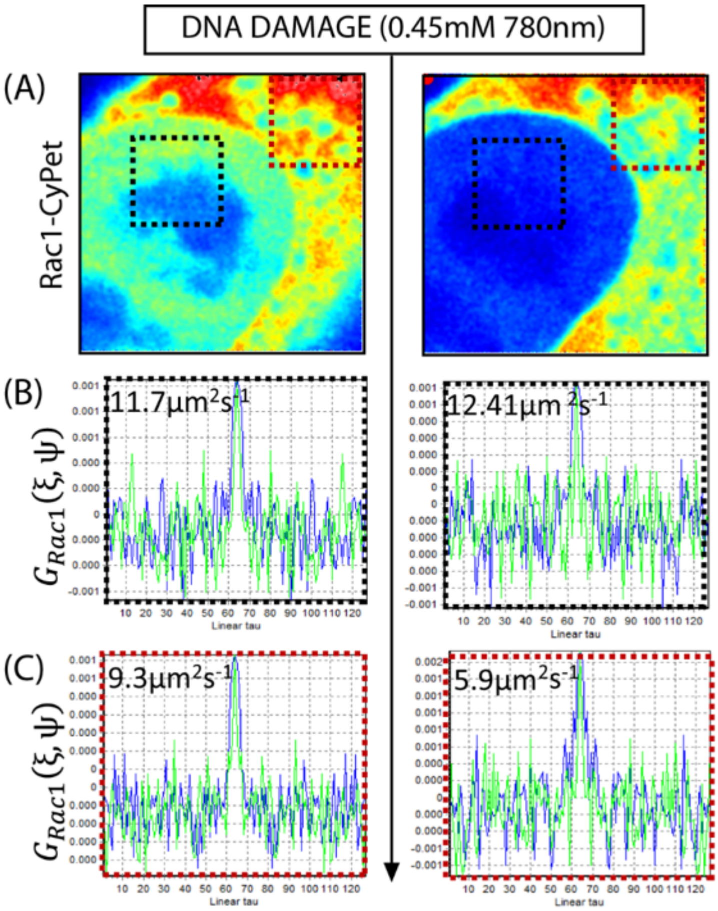

FIGURE S9: RICS analysis of inactive Rac1 diffusion in the nucleus versus cytoplasm, before and after DNA damage (data from experiment presented in Figure 2). (A) Intensity image of an NIH3T3 nucleus transiently transfected with Rac1-CyPet (donor) before (left) and immediately after (right) induction of DNA damage. (B) RICS analysis of Rac1-CyPet diffusion in the nucleus reveals that DNA damage slightly increases Rac1-CyPet mobility. (C) RICS analysis of Rac1-CyPet diffusion in the cytoplasm reveals that DNA damage slows down Rac1-CyPet mobility, despite no FRET or association with PBD-Ypet being observed in this sub-cellular compartment.

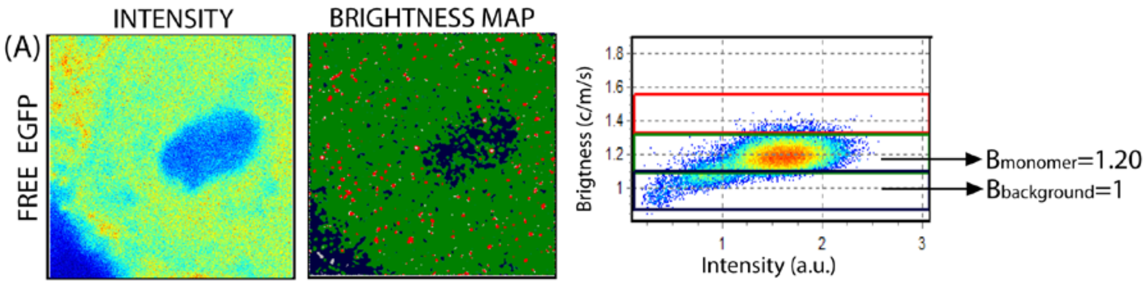

FIGURE S10: Calibration of the monomeric brightness of EGFP to enable extrapolation of the expected apparent brightness of importin-α-EGFP oligomers. (A) Brightness analysis of free EGFP within the nucleus and cytoplasm of an NIH3T3 cell reveals the monomeric distribution of this fluorescent protein to be centred at B=1.20, after correction for the analogue detector (brightness of the background returned to B=1). Thus the molecular brightness of EGFP is 0.20 and we can extrapolate that the apparent brightness of an importin-α-EGFP monomer would be (1+1*0.2)=1.2, a Rac1-CyPet dimer would be (1+0.2*2)=1.4, a Rac1-CyPet trimer would be (1+0.2*3)=1.6 and so on.
